# Multicenter analysis on the value of standard (chemo)radiotherapy in elderly patients with locally advanced adenocarcinoma of the esophagus or gastroesophageal junction

**DOI:** 10.1186/s13014-024-02414-9

**Published:** 2024-03-04

**Authors:** Tilman Bostel, Eirini Nikolaidou, Daniel Wollschläger, Arnulf Mayer, Justus Kaufmann, Anne Hopprich, Alexander Rühle, Anca-Ligia Grosu, Jürgen Debus, Christian Fottner, Markus Moehler, Peter Grimminger, Heinz Schmidberger, Nils Henrik Nicolay

**Affiliations:** 1 Department of Radiation Oncology, University Medical Center Mainz, Langenbeckstraße 1, 55131 Mainz, Germany; 2 German Cancer Consortium (DKTK) Partner Site Mainz, German Cancer Research Center (Dkfz), Heidelberg, Germany; 3 Radiological Institute Dr. Von Essen, Koblenz, Germany; 4 Department of Radiation Oncology, Charité—University Medicine Berlin, corporate member of Freie Universität Berlin and Humboldt-Universität Zu Berlin, Berlin, Germany; 5 German Cancer Consortium (DKTK), Partner Site Berlin, German Cancer Research Center (DKFZ), Heidelberg, Germany; 6 Institute of Medical Biostatistics, Epidemiology and Informatics (IMBEI), University Medical Center Mainz, Mainz, Germany; 7 Department of Radiation Oncology, Peter MacCallum Cancer Centre, Melbourne, VIC Australia; 8 Department of Radiation Oncology, University of Freiburg – Medical Center, Freiburg, Germany; 9 German Cancer Consortium (DKTK), Partner Site Freiburg, German Cancer Research Center (DKFZ), Heidelberg, Germany; 10 Department of Radiation Oncology, University Hospital of Heidelberg, Heidelberg, Germany; 11 German Cancer Consortium (DKTK), Partner Site Heidelberg, German Cancer Research Center (DKFZ), Heidelberg, Germany; 12 Department of Internal Medicine I, University Medical Center Mainz, Mainz, Germany; 13 Department of General, Visceral and Transplant Surgery, University Medical Center Mainz, Mainz, Germany; 14 Department of Radiation Oncology, University of Leipzig Medical Center, Leipzig, Germany; 15 Cancer Center Central Germany (CCCG), Leipzig, Germany

**Keywords:** Esophageal cancer, Elderly patients, Adenocarcinoma, Gastroesophageal Junction tumor, AEG, Chemoradiation, Radiotherapy

## Abstract

**Background:**

To assess the tolerability and oncological results of chemoradiation in elderly patients with locally advanced adenocarcinoma of the esophagus or gastroesophageal junction.

**Methods:**

This multi-center retrospective analysis included 86 elderly patients (≥ 65 years) with esophageal or gastroesophageal junction adenocarcinoma (median age 73 years; range 65–92 years) treated with definitive or neoadjuvant (chemo)radiotherapy. The treatment was performed at 3 large comprehensive cancer centers in Germany from 2006 to 2020. Locoregional control (LRC), progression-free survival (PFS), distant metastasis-free survival (DMFS), overall survival (OS), and treatment-associated toxicities according to CTCAE criteria v5.0 were analyzed, and parameters potentially relevant to patient outcomes were evaluated.

**Results:**

Thirty-three patients (38%) were treated with neoadjuvant chemoradiation followed by surgery, while the remaining patients received definitive (chemo)radiation. The delivery of radiotherapy without dose reduction was possible in 80 patients (93%). In 66 patients (77%), concomitant chemotherapy was initially prescribed; however, during the course of therapy, 48% of patients (n = 32) required chemotherapy de-escalation due to treatment-related toxicities and comorbidities. Twenty-nine patients (34%) experienced higher-grade acute toxicities and 14 patients (16%) higher-grade late toxicities. The 2-year LRC, DMFS, PFS, and OS amounted to 72%, 49%, 46%, and 52%, respectively. In multivariate analysis, neoadjuvant chemoradiation followed by surgery was shown to be associated with significantly better PFS (p = 0.006), DMFS (p = 0.006), and OS (p = 0.004) compared with all non-surgical treatments (pooled definitive radiotherapy and chemoradiation). No such advantage was seen over definitive chemoradiation. The majority of patients with neoadjuvant therapy received standard chemoradiotherapy without dose reduction (n = 24/33, 73%). In contrast, concurrent chemotherapy was only possible in 62% of patients undergoing definitive radiotherapy (n = 33/53), and most of these patients required dose-reduction or modification of chemotherapy (n = 23/33, 70%).

**Conclusions:**

In our analysis, omission of chemotherapy or adjustment of chemotherapy dose during definitive radiotherapy was necessary for the overwhelming majority of elderly esophageal cancer patients not eligible for surgery, and hence resulted in reduced PFS and OS. Therefore, optimization of non-surgical approaches and the identification of potential predictive factors for safe administration of concurrent chemotherapy in elderly patients with (gastro)esophageal adenocarcinoma is required.

**Supplementary Information:**

The online version contains supplementary material available at 10.1186/s13014-024-02414-9.

## Background

The last decades have shown a notable epidemiologic shift with dramatically rising incidence of esophageal adenocarcinoma (EAC) and adenocarcinoma of the esophagogastric junction (AEG) in Western Europe and North America [1]. This increase in Western countries is attributed to the increasing prevalence of risk factors such as obesity, reflux disease and Barrett’s esophagus [2–5]. EAC and AEG are usually diagnosed at a late stage and are associated with a high mortality [1]. However, over the past 20 years, multimodal treatment approaches including neoadjuvant chemoradiotherapy (CRT) and perioperative chemotherapy have been shown to significantly improve prognosis in locally advanced EACs and AEGs compared with surgical treatment alone [6–10]. The current standard of care for operable patients with locally advanced EACs or AEGs is either perioperative multi-agent chemotherapy or neoadjuvant CRT [10, 11]. Furthermore, immune checkpoint inhibitors and anti-HER2 therapies have emerged in recent years as options for the treatment of locally advanced EACs and AEGs with the potential to further improve preoperative therapy [12, 13]. In patients who are inoperable or refuse surgery, definitive CRT may be offered as an alternative curative treatment option [14–16]. Despite significant advances in treatment, the outcome of patients with locally advanced (gastro)esophageal adenocarcinoma still remains unsatisfactory, with 5-year overall survival (OS) rates of only 25% [17].

Due to the increasing life expectancy of the population in Western countries, an increase in elderly patients diagnosed with EACs and AEGs can be observed. The treatment of elderly patients is often challenging, as treatment decisions depend on shifting patient priorities, comorbidities, chronological age and performance status [18, 19]. The lack of or limited inclusion of elderly patients in landmark clinical trials that have investigated the role of CRT in esophageal cancer makes extrapolation of trial data to the older population problematic [10, 15]. To date, there is no uniformly accepted definition of elderly patients; however, most studies specify an age between 65 and 70 years as the threshold for elderly people [20, 21]. For many older patients with locally advanced EACs and AEGs who are no longer amenable to surgical resection, definitive radiotherapy (RT) with or without concurrent chemotherapy remains the only curative treatment option [21]. Despite the proven benefits of concomitant CRT in the neoadjuvant or definitive setting, CRT may lead to serious adverse effects, especially in the presence of comorbidities or poor performance status prior to initiation of treatment.

To date, the available evidence with regard to the benefit of standard definitive or neoadjuvant CRT or RT for the treatment of elderly patients with locally advanced esophageal cancer is scarce and mainly based on retrospective studies [20–24]; to the best of our knowledge, all of these studies included both squamous cell and adenocarcinomas. However, treatment results and the benefit of CRT are considerably different in the two tumor entities. Therefore, this retrospective study aimed to analyze the toxicity profile and oncologic outcomes in a multicenter cohort specifically in elderly patients with locally advanced adenocarcinoma of the esophagus or esophagogastric junction treated with neoadjuvant or definitive (chemo)radiotherapy. We also investigated potential prognostic factors associated with unfavorable treatment response to guide treatment decisions in this vulnerable patient population.

## Methods

### Patients

In this retrospective multi-center study, medical records of 86 elderly patients with histologically confirmed EAC or AEG were reviewed. Inclusion criteria were an age of at least 65 years, no evidence of distant metastases at time of treatment initiation and definitive or neoadjuvant treatment with RT with or without concomitant chemotherapy. Treatment was performed at the University Hospitals of Mainz, Freiburg and Heidelberg between 2006 and 2020. Demographic, clinical and pathological data were collected from electronic medical records, pathology reports and the cancer registries of participating centers. Staging of (gastro-)esophageal carcinomas was based on the versions of the TNM classification (Union for International Cancer Control [UICC]) and the clinical stages of the American Joint Committee on Cancer (AJCC) that were current at the time of first diagnosis (i.e., 6th, 7th or 8th edition of the UICC-AJCC TNM classification). This analysis was approved by the independent ethics committees of the medical faculties of the universities of Mainz (no reference number), Freiburg (reference no. 275/18) and Heidelberg (reference no. S-040/2018).

### Treatment groups

The majority of patients had locally advanced tumors that were treated with either definitive RT with or without concurrent chemotherapy or neoadjuvant CRT followed by surgical resection. Radiation treatment planning was performed using either intensity-modulated radiotherapy (IMRT) or conventional 3D conformal radiotherapy (3D-CRT). All treatment decisions were based on the recommendations of a multidisciplinary tumor board.

Neoadjuvant CRT followed by surgical resection was performed in 33 patients with a median total dose of 41.4 Gy (range 21.6–48.3 Gy) and median single doses of 1.8 Gy (range 1.8–2.0 Gy). Only two of the preoperatively treated patients received sequential or simultaneous dose escalation to the macroscopic tumor (cumulative doses of 41.4–48.3 Gy, single doses 1.8–2.1 Gy). In 6 patients, no surgery was performed after neoadjuvant CRT due to newly detected distant metastases in re-staging (n = 2), worsening of the patient's performance status (n = 2) or patient's refusal of surgery (n = 2). A further 2 patients were switched from the planned neoadjuvant to definitive CRT with consecutive increases in radiation and chemotherapy as they refused surgery. All patients with initial neoadjuvant therapy and no subsequent surgery were assigned to the definitive RT group for the analyses. The approaches to neoadjuvant CRT used until 2012 differed between participating centers (see Additional file 1: Table S1). Starting in 2013, neoadjuvant treatment in all participating centers was performed according to the protocol used for the CROSS trial. The CROSS regimen included RT to a total dose of 41.4 Gy with single doses of 1.8 Gy and concurrent use of paclitaxel (50 mg/m^2^) and carboplatin (AUC of 2 mg/ml/min) on days 1, 8, 15, 22, and 29 [6].

A total of 53 patients received definitive CRT or RT. Primary tumors, lymph node metastases and the elective regional lymphatic drainage area were treated to a median total dose of 50 Gy (range 25.2–60.0 Gy) using median single doses of 1.8 Gy (range 1.8—3.0 Gy). The majority of patients (n = 39, 74%) received dose escalation to the macroscopic tumor tissue by using simultaneous integrated or sequential boost concepts (median total dose 9.0, range 3.6–14.4 Gy; median single dose 2.0 Gy, range 1.8–2.1 Gy; n = 35, 66%) and/or brachytherapy boost (median total dose 15.0, range 8.0–18.0 Gy; median single dose 5.0 Gy, range 4.0–6.0 Gy; n = 13, 25%). The median cumulative dose was 54 Gy (range 25.2–66.0 Gy). Different chemotherapy regimens were applied in combination with definitive RT, as outlined in Additional file 2: Table S2.

Patient eligibility for RT and concomitant chemotherapy was assessed at baseline. Reasons for discontinuation or reduction of RT and concomitant chemotherapy were taken from patient records. For this analysis, we defined combinations of cisplatin or oxaliplatin and infusional 5-fluorouracil (5-FU), carboplatin and paclitaxel and mitomycin C and 5-FU as standard chemotherapy regimens. Mono-chemotherapy regimens with 5-FU or capecitabine or dose reduction of chemotherapy during radiation treatment were defined as chemotherapy modifications. In addition, we defined full-dose RT and full-dose chemotherapy as administration of both treatment modalities without interruption, dose reduction, or modification.

### Target volume definition

Primary gross tumor volume (GTV) and lymph node GTV(s) were defined based on planning computed tomography (CT) and additional imaging studies, including contrast-enhanced CT, positron emission tomography/computed tomography (PET/CT), endosonography, and endoscopy with clipping of oral and aboral tumor borders, if available. The clinical target volume (CTV) for the primary tumor was determined by adding safety margins of 3–5 cm longitudinally and 1–2 cm axially, while CTV(s) for metastatic lymph nodes were generated by adding a safety distance of 1 cm to the GTVs to respect microscopic tumor spread. Elective areas of regional lymphatic drainage were regularly integrated into the total CTV. The CTV was adjusted for anatomic barriers such as bone, lung, or heart and, for distal tumors, the stomach. The planning target volume (PTV) included the CTV and an additional craniocaudal and lateral safety margin of 0.5–1.0 cm to compensate for internal and setup variations.

### Oncologic outcomes and toxicity

All patients received regular follow-up at 3- to 6-month intervals, including clinical and endoscopic examinations and CT staging. If locoregional or distant tumor recurrence was suspected on CT or endoscopy, further diagnostic workup was performed. For all survival analyses, time at risk started with the completion of RT, and ended with the occurrence of an event or at the date of last follow-up, whichever occurred first. For locoregional control (LRC), an event was defined as progression of the primary tumor or new-onset or advanced locoregional lymph node metastases. For distant metastasis-free survival (DMFS), an event was defined as the occurrence of distant metastases or death from any cause. For progression-free survival (PFS), an event was defined as diagnosis of tumor progression at any site or death from any cause. For overall survival (OS), an event was defined as death from any cause. Survival data were obtained from the respective cancer registries. Acute and chronic adverse events were extracted from the patient records and graded according to the CTCAE criteria (version 5.0).

### Statistical analysis

Survival after RT was analyzed using the Kaplan–Meier method, with the log-rank test to determine statistical significance. Multivariable analyses were performed using the Cox proportional hazards model and associated Wald tests to identify predictors of LRC, DMFS, PFS and OS after RT. Since chemotherapy was sometimes completed after completion of RT, tests involving completion of chemotherapy as a predictor were based on a Cox model with time-varying covariates to avoid immortal-time bias. All statistical analyses were performed using R software, version 4.1.3 (R Core Team 2022, Vienna, Austria), and *p*-values of *p* < 0.05 were considered statistically significant.

## Results

A total of 86 elderly patients with a median age of 73 years (range 65–92 years) and histologically confirmed EAC or AEG were included in this retrospective multi-center analysis. Most patients were male (n = 72, 84%). According to the consensus definition of the United States National Institute of Aging, the study population was subdivided into the following 3 age groups: “young olds” (65 to 74 years), “older olds” (75 to 84 years) and “oldest olds” (≥85 years). In our study population, the majority of patients belonged to the "young old" subgroup (n = 50, 58%), whereas the proportion of patients classified as "older old" and "oldest old" amounted to 33% (n = 28) and 9% (n = 8), respectively. The majority of patients exhibited a good performance status before treatment, with 73 patients (85%) having ECOG (Eastern Cooperative Oncology Group) scores of 0 or 1. The majority of adenocarcinomas was localized in the distal thoracic segment of the esophagus or at the esophagogastric junction (n = 64, 74%) and were moderately or poorly differentiated (52% and 40%, respectively). Most patients suffered from locally advanced disease at the time of diagnosis. Seventy-two patients (84%) suffered from locally advanced cT3/4 tumors (cT3/4) and 68 patients (79%) demonstrated lymphogenic tumor spread on imaging. For the detailed information on patient and tumor characteristics, please refer to Table 1.

**Table 1:** Tumor and patient characteristics at baseline

Variable	Value	n	%
Gender	male	72	83.7
	female	14	16.3
Age	65–74 years	50	58.1
	75–84 years	28	32.6
	≥ 85 years	8	9.3
ECOG	0	30	34.9
	1	43	50.0
	2	13	15.1
Localization (distance from incisors)	Cervical (15–18 cm)	1	1.2
	Upper thoracic (18–24 cm)	2	2.3
	Middle thoracic (24–32 cm)	19	22.1
	Lower thoracic (32–approximate 40 cm)	64	74.4
cT-stage	T1	2	2.3
	T2	12	14.0
	T3	62	72.1
	T4	10	11.6
cN-stage	N0	18	20.9
	N +	68	79.1
M-stage	M0	86	100
	M1	0	0
AJCC-stage	1	0	0
	2	7	8.1
	3	58	67.4
	4a	21	24.4
Grading	G1	2	2.3
	G2	45	52.3
	G3	32	37.2
	G4	2	2.3
	Gx	5	5.8
CCI	≤ 5	23	26.7
	> 5	62	72.1
	NA	1	1.2

Staging of the esophageal or gastroesophageal adenocarcinomas was based on the versions of the TNM classification (Union for International Cancer Control [UICC]) and the clinical stages of the American Joint Committee on Cancer (AJCC) that were current at the time of first diagnosis (i.e., 6th, 7th or 8th edition of the UICC-AJCC TNM classification)

*ECOG* Eastern Cooperative Oncology Group, *AJCC* American Joint Committee on Cancer, *CCI* Charlson Comorbidity Index, *NA* not analyzable

Thirty-three patients (38%) received neoadjuvant concomitant chemoradiation followed by surgical resection. Ninety-four percent of surgical patients had a good ECOG performance score (ECOG 0, n = 16/32, 50%; ECOG 1, n = 14/32, 44%) prior to neoadjuvant treatment. Twenty-one of these patients (64%) were treated with carboplatin and paclitaxel, 11 patients (33%) with cisplatin and 5-FU, and one patient with 5-FU alone (3%). In 3 patients (9%), RT had to be discontinued prematurely due to complications (gastric bleeding, pneumonia) or therapy-related side effects with consecutive deterioration of the general condition (odynophagia). Concomitant chemotherapy was reduced or modified in 9 patients (27%) undergoing neoadjuvant CRT due to deteriorating performance status, acute toxicities or treatment complications (gastric perforation). Overall, 28 of neoadjuvantly treated patients (85%) were able to receive more than 80% of the prescribed chemotherapy dose. Furthermore, in the neoadjuvant treatment setting, adherence to CRT with carboplatin/paclitaxel (CROSS regimen) was higher compared to CRT with cisplatin/5-FU (76% [n = 16/21 patients] vs. 64% [n = 7/11 patients]).

Fifty-three patients (62%) were treated with definitive RT, of whom 33 patients (62%) received concurrent CRT and 1 patient received concurrent EGFR receptor antibody (cetuximab). Prior to definitive RT or CRT, 85% of patients had a good ECOG performance score (ECOG 0, n = 9/27, 33%; ECOG 1, n = 14/32, 52%). Different chemotherapy regimens were administered during definitive treatment, including cisplatin/5-FU (n = 20, 38%), carboplatin/paclitaxel (n = 7, 13%), oxaliplatin/5-FU (n = 3, 6%), mitomycin C/5-FU (n = 1, 2%), and 5-FU alone (n = 2, 6%). Fifty patients (94%) received full dose definitive RT, and 30 patients (91%) completed concomitant chemotherapy as initially prescribed. The reasons for premature discontinuation of RT were acute toxicities, severe complications (tracheoesophageal fistula, tumor bleeding, and infectious diseases), exacerbation of existing comorbidity (heart failure), and deteriorating patient performance status. The chemotherapy dose was reduced due to treatment-related toxicities, concomitant diseases (mostly renal failure), and an allergic reaction in one patient. The full treatment regimen of definitive chemoradiotherapy, including all planned concomitant and adjuvant chemotherapy courses, could be administered in only 10 patients (30%) due to treatment-related toxicities. In 14 patients (42%), more than 80% of the originally prescribed chemotherapy dose was administered in the definitive treatment setting. Furthermore, adherence to definitive CRT with carboplatin/paclitaxel was higher compared with definitive CRT with cisplatin/5-FU (43% [n = 3/7 patients] vs. 25% [n = 5/20 patients]).

In the overall study population, the most common chemotherapy regimens administered concurrently with RT were carboplatin/paclitaxel and cisplatin/5-FU. Comparison of carboplatin/paclitaxel (28/66 patients, 42%) and cisplatin/5-FU (31/66 patients, 47%) demonstrated a better tolerability of carboplatin/paclitaxel: The full dose of carboplatin/paclitaxel or cisplatin/5-FU could be administered in 68% (n = 19/28 patients) and 39% of patients (n = 12/31 patients), respectively.

Overall, 18 patients (21%) required bougienage and 16 patients (19%) required stenting due to esophageal stenosis after treatment.

### Treatment outcome

For the entire cohort, the 1-, 2-, and 5-year LRC amounted to 81.9% (95% CI 73.1%-91.8%), 72.1% (95% CI 61.4%-84.6%), and 61.5% (95% CI 48.0%-78.6%). The 1-, 2-, and 5-year DMFS was 64.4% (95% CI 54.6%-75.9%), 48.9% (95% CI 38.8%-61.7%), and 23.5% (95% CI 14.8%–37.5%).

PFS after 1, 2 and 5 years was 63.3% (95% CI 53.3%–75.2%), 46.2% (95% CI 36.0%–59.2%) and 24.1% (95% CI 15.3%–38.1%), and the corresponding OS amounted to 72.9% (95% CI 63.8%–83.3%), 52.3% (95% CI 42.2%–64.9%) and 22.8% (95% CI 14.0%–37.2%), respectively. A detailed summary of the recurrence patterns is given in Additional file 3: Table S3.

**Fig. 1:**
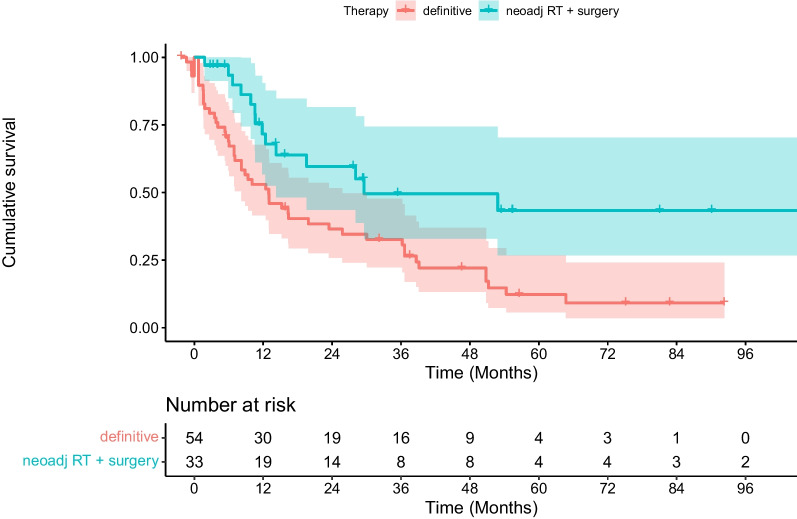
Kaplan–Meier estimate of progression-free survival (PFS) after radiotherapy stratified by neoadjuvant chemoradiation followed by surgical resection vs. definitive treatment with (chemo)radiation. PFS was significantly better for patients who received neoadjuvant treatment (*p* = 0.01, log-rank test)

**Fig. 2:**
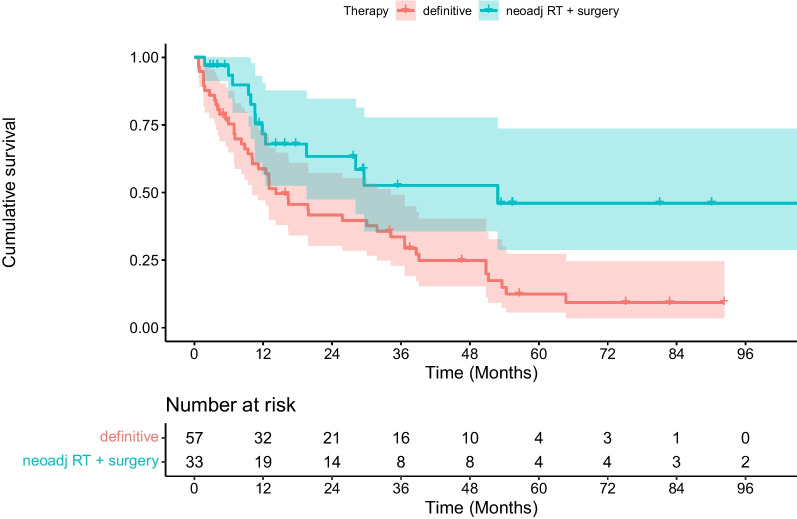
Kaplan–Meier estimate of distant metastasis-free survival (DMFS) after radiotherapy stratified by neoadjuvant chemoradiation followed by surgical resection vs. definitive treatment with (chemo)radiation. DMFS was significantly better for patients who received neoadjuvant treatment (*p* = 0.009, log-rank test)

**Fig. 3:**
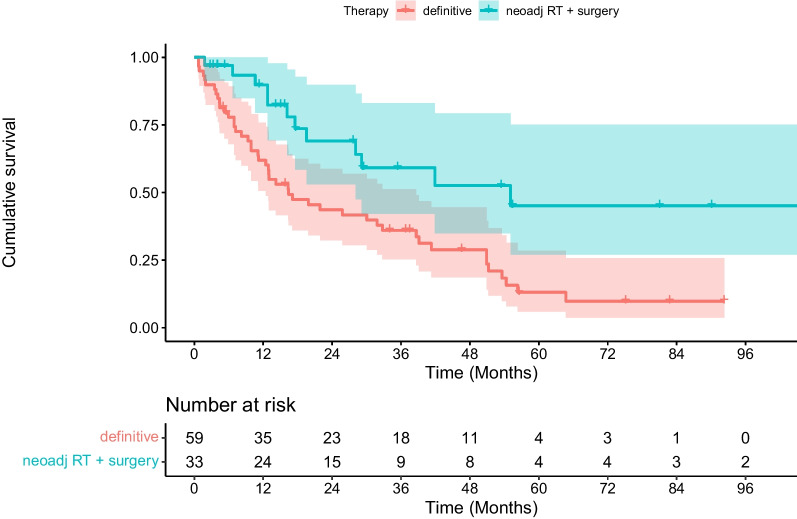
Kaplan–Meier estimate of overall survival (OS) after radiotherapy stratified by neoadjuvant chemoradiation followed by surgical resection vs. definitive treatment with (chemo)radiation. OS was significantly better for patients who received neoadjuvant treatment (*p* = 0.004, log-rank test)

In multivariate analysis, neoadjuvant chemoradiation followed by surgical tumor resection was shown to be associated with significantly better PFS (*p* = 0.006), DMFS (*p* = 0.006) and OS (*p* = 0.004) compared with definitive (chemo)radiotherapy (see Table 2, 3, 4, 5, 6, 7, 8, and Figs. 1, 2, 3). In contrast, age, gender, performance status, comorbidities (Charlson Comorbidity Index), localization of the primary tumor, tumor stage (T), metastatic nodal spread (N stage), disease stage according to the Union for International Cancer Control (UICC), RT adherence (complete vs. incomplete administration), applied radiation dose, and administration of full-dose systemic therapy (vs. no or modified systemic therapy) were not associated with significantly better PFS, DMFS and OS (see Tables 2, 3, 4). In our analysis, older-olds and oldest-olds patients showed significantly better LRC compared with young-olds patients (*p* = 0.01, see Tables 5 and 9).

**Table 2:** Univariate analyses of potential prognostic factors for overall survival (OS)

Factors	OS at 1 year (%)	OS at 2 years (%)	OS at 5 years (%)	p-value
Age				
65–74 years	72	53	22	
75–84 years	78	58	24	
≥ 85 years	60	30	30	0.60
Gender				
Female	79	71	30	
Male	72	48	21	0.10
ECOG score				
0–1	72	54	25	
2–3	77	46	-	0.20
Charlson Comorbidity Index				
≤ 5	64	64	29	
> 5	76	48	19	0.20
Clinical tumor classification (cT)				
1	50	-	-	
2	83	50	17	
3	74	56	27	
4	60	33	17	0.70
Clinical lymph node classification (cN)				
Nodal negative (N0)	75	54	18	
Nodal positive (N +)	73	52	24	0.80
Tumor stage (AJCC)				
1–3	71	52	23	
4a	80	52	19	0.70
Localization of the primary tumor				
15–32 cm distance from the incisors	76	66	32	
33 cm distance from the incisors until gastro-esophageal junction	72	48	19	0.20
Administration of full-dose RT				
yes	33	17	-	
no	76	55	23	0.10
Cumulative dose of RT (EQD2)				
≤ 50 Gy	72	49	31	
> 50 Gy	75	57	17	0.70
Administration of non-modified, full-dose chemotherapy				
yes	81	66	36	
no	68	45	18	0.17
Treatment concept				
Neoadjuvant CRT followed by surgical resection	90	69	45	
Definitive RT / CRT	63	43	14	**0.004**

Bold values significant p-values

*ECOG* Eastern Cooperative Oncology Group, *AJCC* American Joint Committee on Cancer, *RT* radiotherapy, CRT chemoradiotherapy

**Table 3:** Univariate analyses of potential prognostic factors for progression-free survival (PFS)

Factors	PFS at 1 year (%)	PFS at 2 years (%)	PFS at 5 years (%)	p-value
Age				
65–74 years	70	43	23	
75–84 years	79	60	23	
≥ 85 years	63	47	31	0.60
Gender				
Female	71	50	32	
Male	72	49	20	0.30
ECOG score				
0–1	70	50	24	
2–3	85	46	23	0.60
Charlson Comorbidity Index				
≤ 5	59	39	25	
> 5	50	29	6	0.05
Clinical tumor classification (cT)				
1	50	-	-	
2	92	50	17	
3	69	53	30	
4	70	30	10	0.70
Clinical lymph node classification (cN)				
Nodal negative (N0)	82	61	19	
Nodal positive (N +)	70	46	24	0.50
Tumor stage (AJCC)				
1–3	71	51	25	
4a	75	43	17	0.50
Localization of the primary tumor				
15–32 cm distance from the incisors	77	61	45	
33 cm distance from the incisors until gastroesophageal junction	71	45	18	0.05
Administration of full-dose RT				
Yes	67	33	-	
No	73	50	24	0.40
Cumulative dose of RT (EQD2)				
≤ 50 Gy	75	46	29	
> 50 Gy	69	53	19	0.70
Administration of non-modified, full-dose chemotherapy				
Yes	77	60	38	
No	70	43	17	0.79
Treatment concept				
Neoadjuvant CRT followed by surgical resection	86	60	43	
Definitive RT / CRT	64	43	15	**0.01**

Bold values = significant p-values

*ECOG* Eastern Cooperative Oncology Group, *AJCC* American Joint Committee on Cancer, *RT* radiotherapy, *CRT* chemoradiotherapy

**Table 4:** Univariate analyses of potential prognostic factors for distant metastasis-free survival (DMFS)

Factors	DMFS at 1 year (%)	DMFS at 2 years (%)	DMFS at 5 years (%)	p-value
Age				
65–74 years	60	45	23	
75–84 years	73	61	25	
≥ 85 years	60	30	30	0.50
Gender				
Female	71	57	37	
Male	63	47	20	0.20
ECOG score				
0–1	65	50	25	
2–3	62	46	-	0.50
Charlson Comorbidity Index				
≤ 5	62	53	39	
> 5	64	47	19	0.30
Clinical tumor classification (cT)				
1	50	-	-	
2	75	50	17	
3	66	52	28	
4	44	30	15	0.80
Clinical lymph node classification (cN)				
Nodal negative (N0)	75	54	19	
Nodal positive (N +)	62	48	25	0.60
Tumor stage (AJCC)				
1–3	65	48	25	
4a	63	52	14	0.60
Localization of the primary tumor				
15–32 cm distance from the incisors	75	64	44	
33 cm distance from the incisors until gastroesophageal junction	61	44	19	0.10
Administration of full-dose RT				
yes	17	17	-	
no	68	52	24	0.20
Cumulative dose of RT (EQD2)				
≤ 50 Gy	59	46	32	
> 50 Gy	71	53	17	0.70
Administration of non-modified, full-dose chemotherapy				
yes	67	59	38	
no	63	44	18	0.26
Treatment concept				
Neoadjuvant CRT followed by surgical resection	72	63	46	
Definitive RT / CRT	60	41	14	**0.009**

Bold values = significant p-values

*ECOG* Eastern Cooperative Oncology Group, *AJCC* American Joint Committee on Cancer, *RT* radiotherapy, *CRT* chemoradiotherapy

**Table 5:** Univariate analyses of potential prognostic factors for locoregional control (LRC)

Factors	LRC at 1 year (%)	LRC at 2 years (%)	LRC at 5 years (%)	p-value
Age				
65–74 years	74	61	44	
75–84 years	92	85	85	
≥ 85 years	100	100	100	**0.02**
Gender				
Female	83	56	46	
Male	82	77	64	0.20
ECOG score				
0–1	80	74	68	
2–3	91	66	-	0.50
Charlson Comorbidity Index				
≤ 5	84	71	59	
> 5	81	72	64	0.90
Clinical tumor classification (cT)				
1	100	-	-	
2	90	77	51	
3	81	73	65	
4	71	57	57	0.90
Clinical lymph node classification (cN)				
Nodal negative (N0)	92	75	63	
Nodal positive (N +)	80	72	63	0.80
Tumor stage (AJCC)				
1–3	80	72	62	
4a	89	70	56	1.00
Localization of the primary tumor				
15–32 cm distance from the incisors	89	82	70	
33 cm distance from the incisors until gastroesophageal junction	80	68	59	0.50
Administration of full-dose RT				
Yes	75	75	-	
No	83	72	61	0.90
Cumulative dose of RT (EQD2)				
≤ 50 Gy	81	78	69	
> 50 Gy	83	66	56	0.40
Administration of non-modified, full-dose chemotherapy				
Yes	81	77	55	
No	83	69	65	0.72
Treatment concept				
Neoadjuvant CRT followed by surgical resection	88	84	73	
Definitive RT/CRT	78	64	56	0.10

Bold values = significant p-values

*ECOG* Eastern Cooperative Oncology Group, *AJCC* American Joint Committee on Cancer, *RT* radiotherapy, *CRT* chemoradiotherapy

**Table 6:** Cox regression analysis of clinical parameters regarding ***overall survival*** after definitive or neoadjuvant CRT/RT

Factors	HR	CI 95%	p-value
Age	0.98	0.94–1.03	0.47
Neoadjuvant CRT followed by surgery vs. definitive RT/CRT	0.36	0.18–0.73	**0.004**

*CRT* chemoradiotherapy, *RT* radiotherapy

Bold values = significant p-values

**Table 7:** Cox regression analysis of clinical parameters regarding ***progression-free survival*** after definitive or neoadjuvant CRT/RT

Factors	HR	CI 95%	p-value
Age	0.97	0.92–1.02	0.23
Neoadjuvant CRT followed by surgery vs. definitive RT / CRT	0.39	0.19–0.77	**0.006**

Bold values = significant p-values

*CRT* chemoradiotherapy, *RT* radiotherapy

**Table 8:** Cox regression analysis of clinical parameters regarding ***distant metastasis-free survival*** after definitive or neoadjuvant CRT/RT

Factors	HR	CI 95%	p-value
Age	0.97	0.93–1.02	0.29
Neoadjuvant CRT followed by surgery vs. definitive RT / CRT	0.38	0.19–0.76	**0.006**

*CRT* chemoradiotherapy, *RT* radiotherapy

Bold value = significant p-value

**Table 9:** Cox regression analysis of clinical parameters regarding ***locoregional control*** after definitive or neoadjuvant CRT/RT

Factors	HR	CI 95%	p-value
Age	0.89	0.81–0.97	**0.01**

Bold value = significant p-value

*CRT* chemoradiotherapy, *RT* radiotherapy

Survival outcomes and locoregional tumor control for patients who received definitive RT or definitive CRT were considered separately to reduce bias when comparing to patients who received neoadjuvant CRT. When comparing definitive CRT (n = 33 patients) and neoadjuvant CRT (n = 33 patients), no relevant differences in LRC, DMFS and PFS were detectable. OS was better for patients with neoadjuvant CRT followed by resection as compared to definitive CRT without reaching statistical significance in the univariate analysis (*p*-value 0.06). The results for the differentiated group analysis are summarized in detail in Table 10.

**Table 10:** Univariate comparison of the patients treated for (gastro)esophageal adenocarcinoma with neoadjuvant CRT vs. definitive CRT

Oncological endpoints	Neoadjuvant CRT (n = 33 patients)	Definitive CRT (n = 33 patients)	p—value
LRC			
at 1 year in % (95% CI)	88.2 (76.6–100)	78.5 (64.6–95.4)	
at 2 years in % (95% CI)	83.8 (70.4–99.8)	65.3 (49.1–86.9)	
at 5 years in % (95% CI)	73.4 (53.6–100)	59.9 (42.9–83.5)	0.2
PFS			
at 1 year in % (95% CI)	71.6 (56.7–90.5)	67.7 (53.1 – 86.4)	
at 2 years in % (95% CI)	59.6 (43.6–81.6)	47.8 (33.0 – 69.3)	
at 5 years in % (95% CI)	43.3 (26.7–70.3)	17.0 (7.3 – 39.9)	0.1
DMFS			
at 1 year in % (95% CI)	71.6 (56.7–90.5)	71.4 (57.2 – 89.1)	
at 2 years in % (95% CI)	63.4 (47.4–84.6)	54.7 (39.6 – 75.5)	
at 5 years in % (95% CI)	46.1 (28.8–73.6)	16.9 (7.2 – 39.8)	0.1
OS			
at 1 year in % (95% CI)	89.8 (79.4–100)	72.3 (58.4 – 89.5)	
at 2 years in % (95% CI)	69.0 (53.1–89.8)	56.1 (41.2 – 76.4)	
at 5 years in % (95% CI)	45.1 (27.1–75.1)	17.8 (7.7 – 41.3)	0.06

*LRC* Locoregional control, *PFS* Progression-free survival, *DMFS* Distant metastasis-free survival, *OS* Overall survival

### Treatment-related toxicities

Acute severe toxicities (CTCAE grade 3) occurred in 33% of patients (28/86 patients) during the course of (chemo)irradiation, with hematologic adverse events (n = 13 patients, 15%), new-onset or progressive dysphagia and odynophagia with consecutive weight loss, and increasing stenosis of the esophageal lumen (n = 9 patients, 10%) being the most common adverse events. One patient (1%) had a fatal outcome due to complications resulting from a trachea-esophageal fistula (CTCAE grade 5). In the neoadjuvant setting, higher-grade acute toxicities were observed in 27% of patients (n = 9/33 patients; hematologic: n = 1 patient, 3%; non-hematologic: n = 8/33 patients, 24%) with consecutive modification and dose reduction of concomitant CRT in all affected patients. In the patient cohort with definitive RT or CRT, higher-grade acute toxicities were documented in 36% of patients (n = 19/53 patients; hematologic: n = 10/53 patients, 19%; non-hematologic: n = 9/53 patients, 17%), and dose reduction of RT or CRT was required in 70% of patients (n = 23/33 patients).

Higher-grade late toxicities (CTCAE grade 3) were diagnosed in 14 patients (16%), with the onset of esophageal stenoses (n = 13 patients) being most dominant. The detailed toxicity profile of radiation or chemoradiation treatment is summarized in Table 11.

**Table 11:** Summary of (chemo)radiotherapy-associated toxicities ≥ grade 3 according to CTCAE version 5.0

Variable	Value
Acute toxicities–no. (%)	29 (33.7)
Hematological side effects – no. (%)	13 (15.1)
Dysphagia / odynophagia – no. (%)	9 (10.5)
Esophageal stenosis – no. (%)	3 (3.5)
Mucositis – no. (%)	1 (1.2)
Acute renal failure – no. (%)	1 (1.2)
Tumor bleeding – no. (%)	2 (2.3)
Fistula with consecutive pneumonia – no. (%)	1 (1.2)
Nausea / emesis – no. (%)	1 (1.2)
Lethal event	1 (1.2)
Late toxicities – no. (%)	14 (16.3)
Dysphagia – no. (%)	13 (15.1)
Esophageal stenosis – no. (%)	13 (15.1)
Fistula – no. (%)	2 (2.3)

*CTCAE* Common Terminology Criteria of Adverse Events

## Discussion

In our multi-center patient cohort of elderly esophageal adenocarcinoma patients, RT was well tolerated. Overall, 93% of patients received the prescribed RT dose; however, only 77% of patients were initially deemed suitable for concurrent administration of chemotherapy and RT. Of these patients, 95% received standard chemotherapy concurrently to definitive or neoadjuvant RT. Modification or dose reduction of concurrent chemotherapy (including chemotherapy cycles after RT) was required in almost half of patients (48%) due to acute toxicities or deteriorating general condition. Compared with published phase III trials, adherence to CRT was worse in our study [10, 14, 16, 25]; the difference was most pronounced when our results were compared with studies in which neoadjuvant or definitive CRT with carboplatin/paclitaxel or oxaliplatin/5-FU was administered [10, 14, 16]. Our analysis showed that dose reduction or modification of concurrent chemotherapy occurred more frequently in the subgroup of patients with definitive CRT than in the subgroup of patients with neoadjuvant CRT (70% vs. 27%). This result is consistent with the results of prospective studies. For example, simultaneous CRT with 5 weekly doses of carboplatin/paclitaxel and a radiation dose of 41.4 Gy in 23 fractions could be administered in 91% of patients in the CROSS trial, whereas the same chemotherapy regimen with only one additional weekly cycle of chemotherapy and a slightly higher radiation dose (50.4 Gy in 28 fractions) could be administered in only 69% of patients in the standard-dose arm of the ARTDECO trial [10, 14]. However, in our elderly patient population with EACs and AEGs, the better treatment tolerability of neoadjuvant CRT compared with definitive CRT did not translate into a benefit regarding LRC, PFS or DMFS. However, there was a non-significant trend towards better OS in favor of neoadjuvant CRT and consecutive surgery. In contrast, in a large multicenter study of esophageal squamous cell carcinoma, treatment adherence and trimodal therapy with neoadjuvant CRT and surgical tumor resection significantly improved PFS and OS compared with definitive CRT [26]. As in our current analysis, prospective phase III studies failed to demonstrate a statistically significant survival advantage of neoadjuvant CRT over definitive CRT [27, 28]. However, comparing our study results with the results of these prospective phase III studies is problematic because the treatment regimens differed substantially and elderly patients and adenocarcinoma histologies were strongly underrepresented in these trials. Taking this limitation into account, the oncologic outcomes of our elderly patient population, with a median PFS of 19.5 months and a median OS of 28 months, are comparable or even better than in many prospective studies [25, 27, 29]. Our analysis showed a significant advantage of the oldest-old subgroup with respect to LRC. Due to the limited number of patients in this subgroup and the poor 2-year survival, we assume that this result primarily is a statistical artifact.

In our study, the most common chemotherapy regimens administered concurrently with RT were carboplatin/paclitaxel (28/66 patients, 42%) and cisplatin/5-FU (31/66 patients, 47%). In the definitive CRT cohort, a cisplatin / 5-FU regimen was preferentially administered (61%), whereas in the neoadjuvant CRT cohort, the carboplatin / paclitaxel was administered in most patients (64%). Concomitant chemotherapy with carboplatin/paclitaxel proved to be better tolerated than cisplatin/5-FU. In the neoadjuvant and definitive therapy setting, treatment compliance with carboplatin/paclitaxel was considerably higher than with cisplatin/5-FU (68% vs. 39%). Compared with the large phase III landmark trials, treatment tolerance was significantly worse for both the carboplatin/paclitaxel chemotherapy combination as well as cisplatin/5-FU in our elderly patient population [10, 14, 25]. Besides the administered chemotherapy, further possible reasons for the difference in chemotherapy tolerability between the two treatment groups in our study could have been patient-specific and treatment-related factors (e.g., general condition before therapy initiation, comorbidities, acute radiotherapy-related toxicities). For instance, in our analysis, the general condition of the patient cohort with neoadjuvant CRT was slightly better than in the cohort with definitive CRT, consistent with the well-reported decision towards surgical approaches in elderly patients with better overall baseline health.

Severe acute toxicities (CTCAE ≥ III°) occurred in about one third of our study patients (34%), with the most common adverse events being hematologic toxicities and severe esophagitis with consecutive dysphagia and odynophagia. Therefore, before starting therapy, the nutritional status should be quantified and adequate nutrition should be ensured regardless of patient age. Possible measures include the placement of a gastrostomy or jejunostomy tube, in order to avoid unnecessary pauses or modifications of treatment. In the neoadjuvant setting, higher-grade acute toxicities were slightly higher and treatment adherence of our elderly study population was worse than in the CROSS trial (full CRT received: 73% vs. 91% [CROSS study population]) [10]. In the patient cohort with definitive RT or CRT, the treatment adherence was very poor (full CRT received: 30%), but high-grade acute toxicities were documented less frequently than in many phase III landmark trials [14, 16, 25]. As an explanation for this discrepancy, treatment de-escalation in older patients is more readily performed in case of mild-to-moderate acute side effects than in younger patients. Severe late toxicities (CTCAE III°) were reported in 16% of our elderly patients, with dysphagia and esophageal stenoses being the most common side effects, and most of these patients required bougienage or stent implantation. This finding is in line with other studies [15, 26].

In contrast to our results, previous studies have shown that age-related modifications of standard therapy significantly impact the treatment response of tumor diseases [26, 30, 31]. However, despite the high clinical relevance, there is a lack of prospective therapeutic studies with elderly patients only suffering from locally advanced esophageal cancer [32]. To the best of our knowledge, there are no existing prospective trial data on exclusively elderly patients with locally advanced EACs or AEGs. Furthermore, published retrospective studies on (multimodality) therapy of elderly patients with esophageal cancer have predominantly considered mixed patient cohorts with both squamous cell carcinoma and adenocarcinoma histologies [33–36].

Albeit our analysis provides comprehensive data on treatment adherence, toxicity and oncologic outcomes in a large multi-center cohort of elderly EAC and AEG patients undergoing neoadjuvant or definitive (chemo)radiation, it has several limitations due to its retrospective character.

First, detailed information on comorbidities, frailty status, smoking status and clinical data such as laboratory parameters, body mass index and body weight before and during (chemo)radiation may not have been systematically captured. Second, it was not possible to retrospectively assess treatment-related functional adverse events regarding their potential impact on patients' quality of life. Third, a major problem in retrospective assessment of the general health condition is the high variability between different observers [37]; consequently, structured geriatric assessments that encompass many different domains of older patients' lives, such as functional, nutritional, cognitive, psychosocial, and socioeconomic status, could provide a more reliable assessment of patients' performance [38]. For other tumor entities, it has already been shown that geriatric assessment can predict treatment tolerability [39, 40]. Therefore, the predictive potential of geriatric assessments should be further investigated in future prospective trials.

## Conclusion

In summary, our multi-center analysis of 86 elderly patients with adenocarcinoma of the esophagus or esophago-gastric junction could show that half of the chemotherapy-eligible patients required adjustment of chemotherapy due to comorbidities or toxicities. De-escalation of therapy was observed in particular in the subgroup of patients who received definitive (chemo)radiation due to decreased pre-treatment patient performance status and comorbidities. Furthermore, concomitant chemotherapy with carboplatin/paclitaxel proved to be better tolerated than cisplatin/5-FU. Therefore, we recommend carboplatin/paclitaxel as the preferred chemotherapy regimen in combination for both neoadjuvant and definitive CRT in elderly EAC and AEG patients. Future prospective studies should focus on geriatric and comorbidity assessments before therapy and early supportive care to optimize treatment in this vulnerable patient cohort.

### Supplementary Information

**Additional file 1. Table S1.** Chemotherapy regimens concurrent with neoadjuvant radiotherapy.

**Additional file 2. Table S2.** Chemotherapy regimens concurrent with definitive radiotherapy.

**Additional file 3. Table S3.** Recurrence patterns of elderly patients after definitive or neoadjuvant (chemo)radiotherapy.

## Data Availability

All data generated or analysed during the current study are included in this published article and its supplementary information files. The dataset is available from the corresponding author on reasonable request.

## Abbreviations

AEG: Adenocarcinoma of the esophagogastric junction
AJCC: American Joint Committee on Cancer
CRT: Chemoradiotherapy
3D-CRT: 3D conformal radiotherapy
CT: Computed tomography
CTCAE: Common Terminology Criteria for Adverse Events
CTV: Clinical target volume
DMFS: Distant metastasis-free survival
EAC: Esophageal adenocarcinoma
ECOG: Eastern Cooperative Oncology Group
5-FU: 5-Fluorouracil
GTV: Gross tumor volume
IMRT: Intensity-modulated radiotherapy
LRC: Locoregional control
OS: Overall survival
PET/CT: Positron emission tomography/computed tomography
PFS: Progression-free survival
PTV: Planning target volume
RT: Radiotherapy
UICC: Union for International Cancer Control
