# Supplementary material for: Multicenter analysis on the value of standard (chemo)radiotherapy in elderly patients with locally advanced adenocarcinoma of the esophagus or gastroesophageal junction
Source: Radiat Oncol. 2024 Mar 4;19:28. doi: 10.1186/s13014-024-02414-9 (PMC10910868; doi:10.1186/s13014-024-02414-9)
Supplement: Supplementary file 1 — Additional file 1. Table S1. Chemotherapy regimens concurrent with neoadjuvant radiotherapy. [file 13014_2024_2414_MOESM1_ESM.docx]

**Supplemental file 1:**

**Table S1** Chemotherapy regimens concurrent with neoadjuvant radiotherapy

| **Chemotherapy regimens** | **n** | **%** |
| --- | --- | --- |
| Cisplatin (20 mg/m^2^ of body surface area) d1-5 and 5-FU (1000 mg/m^2^ of body surface area) d1-5 at weeks 1 and 5 | 5 | 15.2 |
| Cisplatin (75 mg/m^2^ of body surface area) d7 and 5-FU (15 mg per kilogram of body weight) d1-5 at weeks 1 and 6 | 6 | 18.2 |
| Paclitaxel (50 mg/m^2^ of body surface area) and Carboplatin (area under the curve of 2 mg/ml/min) d1, 8, 15, 22 and 29 | 21 | 63.6 |
| 5-FU alone (1000 mg/m^2^ of body surface area) d1-5 at weeks 1 and 5 | 1 | 3.0 |

**Abbreviations:** 5-FU = 5-fluorouracil, d = day, n = number of patients
