# Supplementary material for: Multicenter analysis on the value of standard (chemo)radiotherapy in elderly patients with locally advanced adenocarcinoma of the esophagus or gastroesophageal junction
Source: Radiat Oncol. 2024 Mar 4;19:28. doi: 10.1186/s13014-024-02414-9 (PMC10910868; doi:10.1186/s13014-024-02414-9)
Supplement: Supplementary file 2 — Additional file 2. Table S2. Chemotherapy regimens concurrent with definitive radiotherapy. [file 13014_2024_2414_MOESM2_ESM.docx]

**Supplemental file 2:**

**Table S2** Chemotherapy regimens concurrent with definitive radiotherapy

| **Chemotherapy regimens** | **n** | **%** |
| --- | --- | --- |
| Cisplatin (75 mg/m^2^ of body surface area) d1 and 5-FU (1000 mg/m^2^ of body surface area) d1-4 at weeks 1, 5, 8 and 11 | 5 | 15.2 |
| Cisplatin (20 mg/m^2^ of body surface area) d1-5 and 5-FU (600 mg/m^2^ of body surface area) d1-5 at weeks 1 and 5 | 4 | 12.1 |
| Cisplatin (20 mg/m^2^ of body surface area) d1-5 and 5-FU (1000 mg/m^2^ of body surface area) d1-5 at weeks 1, 5, 9 and 13 | 11 | 33.3 |
| Paclitaxel (50 mg/m^2^ of body surface area) and Carboplatin (area under the curve of 2 mg/ml/min) d1, 8, 15, 22, 29 and 36 | 7 | 21.2 |
| Oxaliplatin (85mg/m^2^ of body surface area), leucovorin (200 mg/m^2^ of body surface area) and 5-FU (400 mg/m^2^ of body surface area) bolus on day 1 followed by 5-FU (1600 mg/m^2^ of body surface area) 46 hours continuous infusion (FOLFOX) at weeks 1, 3, 5, 7, 9 and 11 | 3 | 9.1 |
| Mitomycin C (10 mg/m^2^ of body surface area) d1 and 5-FU (1000 mg/m^2^ of body surface area) d1-4 at weeks 1, 5, 8 und 11 | 1 | 3.0 |
| Capecitabine or 5-FU alone* | 2 | 6.1 |

* = no informations on dosage and administration.

**Abbreviations:** 5-FU = 5-fluorouracil, d = day, n = number of patients
