# Supplementary material for: Multicenter analysis on the value of standard (chemo)radiotherapy in elderly patients with locally advanced adenocarcinoma of the esophagus or gastroesophageal junction
Source: Radiat Oncol. 2024 Mar 4;19:28. doi: 10.1186/s13014-024-02414-9 (PMC10910868; doi:10.1186/s13014-024-02414-9)
Supplement: Supplementary file 3 — Additional file 3. Table S3. Recurrence patterns of elderly patients after definitive or neoadjuvant (chemo)radiotherapy. [file 13014_2024_2414_MOESM3_ESM.docx]

**Supplemental file 3:**

**Table S3** Recurrence patterns of elderly patients after definitive or neoadjuvant (chemo)radiotherapy.

| **Variable** | **Value** | **n** | **%** |
| --- | --- | --- | --- |
| Locoregional relapse | yes | 21 | 24.4 |
|  | no | 65 | 75.6 |
| Local relapse | yes | 16 | 18.6 |
|  | no | 70 | 81.4 |
| Progressive disease after neoadjuvant or definitive (chemo)radiotherapy | yes | 58 | 67.4 |
|  | no | 28 | 32.6 |
| Distant metastases | yes | 22 | 25.6 |
|  | no | 64 | 74.4 |
| Liver metastases | yes | 7 | 8.1 |
|  | no | 79 | 91.9 |
| Lung metastases | yes | 8 | 9.3 |
|  | no | 78 | 90.7 |
| Bone metastases | yes | 7 | 8.1 |
|  | no | 79 | 91.9 |
| Brain metastases | yes | 1 | 1.2 |
|  | no | 85 | 98.8 |
| Peritoneal carcinomatosis | yes | 2 | 2.3 |
|  | no | 84 | 97.7 |
| Other distant metastases | yes | 3 | 3.5 |
|  | no | 83 | 96.5 |
